# Assessment of trimethylamine-N-oxide at the blood-cerebrospinal fluid barrier: Results from 290 lumbar punctures

**DOI:** 10.17179/excli2020-2763

**Published:** 2020-09-09

**Authors:** Dietmar Enko, Sieglinde Zelzer, Tobias Niedrist, Sandra Holasek, Andreas Baranyi, Wolfgang J. Schnedl, Markus Herrmann, Andreas Meinitzer

**Affiliations:** 1 Clinical Institute of Medical and Chemical Laboratory Diagnostics, Medical University of Graz, Graz, Austria; 2 Institute of Clinical Chemistry and Laboratory Medicine, General Hospital Hochsteiermark, Leoben, Austria; 3 Department of Immunology and Pathophysiology, Medical University of Graz, Otto Loewi Research Center, Graz, Austria; 4 Department of Psychiatry and Psychotherapeutic Medicine, Medical University of Graz, Graz, Austria; 5 Practice for General Internal Medicine, Bruck/Mur, Austria

**Keywords:** biomarkers, blood-cerebrospinal fluid barrier, liquid chromatography-tandem mass spectrometry, trimethylamine-N-oxide

## Abstract

Recently, the microbiome-derived trimethylamine-N-oxide (TMAO) was shown to be present in human cerebrospinal fluid (CSF). However, data on the potential of TMAO crossing the blood-CSF barrier are still lacking. This retrospective study aimed at investigating possible associations between the CSF/serum albumin (Q_ALB_) and TMAO (Q_TMAO_) quotient and evaluating Q_TMAO _values in individuals with and without blood-CSF barrier dysfunction. A total of 290 patients, who underwent diagnostic lumbar puncture with Q_ALB_ and Q_TMAO _determination, were evaluated. Serum and CSF TMAO measurements were performed on a tandem mass spectrometry SCIEX QTRAP 4500 (Applied Biosystems, Framingham, MA, USA) coupled with an Agilent 1260 Infinity HPLC system (Agilent Technologies Santa Clara, CA, USA). Serum and CSF albumin were measured on the Atellica^® ^NEPH 630 system (Siemens Healthineers, Erlangen, Germany). CSF TMAO levels were positively correlated with serum TMAO levels (ρ = 0.709, p < 0.001). The Q_ALB _was significantly associated with the Q_TMAO _(ß-coefficient = 0.312; p < 0.001). A total of 117 patients with blood-CSF barrier dysfunction had significantly higher median (Q1 - Q3) Q_TMAO _values (4.7 (2.8 - 7.5) vs. 3.8 (2.5 - 5.7) x 10^-1^, p = 0.002) compared to 173 individuals with normal blood-CSF barrier function. CSF and serum TMAO concentrations were significantly associated in 290 CSF/serum pairs from lumbar punctures of clinical routine. Q_ALB _showed a relevant influence on Q_TMAO_. Present results indicate that TMAO may cross the blood-CSF barrier.

## Introduction

The small organic molecule trimethylamine-N-oxide (TMAO) is generated in a gut microbiota-dependent way. The gut microbiome metabolizes L-carnitine and phosphatidylcholine containing nutrients (i.e., eggs, cheese, sea salt fish, red meat) to trimethylamine (TMA), which is absorbed into the bloodstream and oxidized by the hepatic flavin mono-oxygenases (FMOs) (Tang et al., 2013[17]; Bennett et al., 2013[1]). Serum TMAO levels depend on multiple factors including diet, gut microbiome composition, liver function and the gut-blood barrier permeability (Ufnal and Pham, 2017[19]).

Several works suggested that TMAO is closely related to neurological disorders (Zhai et al., 2019[25]; Vogt et al., 2018[21]; Xu and Wang, 2016[23]; Janeiro et al., 2018[7]). High serum TMAO concentrations were proposed to predict unfavorable clinical outcomes in patients with acute ischemic stroke and to promote brain aging (Zhai et al., 2019[25]). In a murine cohort, serum TMAO was connected to the process of brain aging (Li et al., 2018[8]). Recently, it was shown that TMAO is also present in the human cerebrospinal fluid (CSF) (Del Rio et al., 2017[4]). Individuals with Alzheimer's disease were observed with elevated CSF TMAO concentrations (Vogt et al., 2018[21]).

It is well known that the CSF analysis is an essential diagnostic tool for neurological diseases. However, the role of TMAO in the brain has not been fully explored yet. The blood-CSF barrier, which is formed by apical tight junctions between the choroid plexus epithelial cells, regulates the permeability of agents from the bloodstream to the CSF (Engelhardt and Sorokin, 2009[5]). The CSF/serum albumin quotient (Q_ALB_) has been widely accepted as a reliable indicator of the blood-CSF barrier function (Reiber and Peter, 2001[13]). Since albumin is not synthesized in the brain, this ratio represents the serum albumin fraction, which diffuses into the CSF. Nevertheless, studies investigating the potential of TMAO crossing the human blood-CSF barrier are not available yet. 

The aim of this retrospective study was to evaluate the albumin and TMAO concentrations in 290 consecutive CSF/serum pairs of lumbar punctures. We assessed possible associations between the CSF/serum albumin (Q_ALB_) and the CSF/serum TMAO (Q_TMAO_) quotient and investigated the Q_TMAO _in individuals sub-grouped by the presence or absence of blood-CSF barrier dysfunction.

## Materials and Methods

### Patient samples

In total, the medical records of 290 consecutive patients, who received diagnostic lumbar puncture with Q_ALB_ and Q_TMAO_ assessment at the Institute of Clinical Chemistry and Laboratory Medicine of the General Hospital Hochsteiermark (Leoben, Austria) in a one year period (2019), were retrospectively examined. Patients < 18 years of age were excluded. Informed consent was obtained from all patients. The ethical approval of this study was provided by the Ethical Committee of the Medical University Graz (Graz, Austria) and carried out with the current version of the declaration of Helsinki.

### Laboratory procedures

CSF and venous blood were taken simultaneously and collected in sterile 2 mL VACUETTE^® ^ Z No Additive and 5 mL VACUETTE^® ^ Z Serum Clot activator tubes (Greiner Bio-one International GmbH, Kremsmünster, Austria). Samples were kept at 4 °C and batch analyzed within one week. The serum (reference range: 3.5 - 5.2 g/dL) and CSF (reference range: ≤ 0.35 g/L) concentrations of albumin were determined by nephelometric method on the Atellica^® ^NEPH 630 system (Siemens Healthineers, Erlangen, Germany). The intra- and inter-day coefficients of variation (CVs) ranged between 2.7 - 3.1 and 1.7 - 3.5 %. The Q_ALB_ was calculated to assess the blood-CSF barrier function (Sindic et al., 2001[16]). According to Reiber et al. (Reiber et al., 2001[9]; Brettschneider et al., 2005[2]), the upper limit of the reference range for Q_ALB _between normal and dysfunctional blood-CSF barrier was determined age-related (5.0 x 10^-3 ^for patients < 15 years, 6.5 x 10^-3 ^for patients < 60 years, and 8.0 x 10^-3 ^for patients ≥ 60 years).

Serum and CSF TMAO were measured using a stable-isotope-dilution assay and high-performance liquid chromatography (HPLC) with electrospray ionization tandem mass spectrometry on a SCIEX QTRAP 4500 triple quadrupole instrument (Applied Biosystems, Framingham, MA, USA) equipped with an Agilent 1260 Infinity HPLC system (Agilent Technologies Santa Clara, CA, USA). The intra- and inter-day CVs ranged between 2.2 - 5.5 and 7.6 - 9.9 %. The serum TMAO reference range (0.98 - 15.5 µmol/L) was calculated according to the literature (Wang et al., 2014[22]).

### Statistical analysis

The Kolmogorov-Smirnov test was performed to calculate data distribution. As all analyzed continuous variables were not normally distributed, they were expressed as medians (Q1 - Q3). To assess potential correlation between two continuous variables the Spearman's rank correlation coefficient (not normally distributed data) was used. Linear regression models were performed to assess the association between variables. The exact Mann-Whitney U test was used for subgroup comparisons. A p-value < 0.05 was considered statistically significant. For statistical analysis, the Analyse-it® software version 4.92 (Analyse-it Software, Ltd., Leeds, United Kingdom) was used.

## Results

### Study population characteristics

The baseline characteristics of the study population are shown in Table 1(Tab. 1). Of all 290 patients with diagnostic lumbar punctures, 145 (50 %) were female, and 145 (50 %) were male. The average age was 53 ± 20 years. 

The median (Q1 - Q3) Q_ALB _was 6.8 (5.0 - 9.9) x 10^-3^. All in all, 117 and 173 individuals were identified with and without blood-CSF barrier dysfunction. The median TMAO CSF and serum concentrations (Q1 - Q3) were 0.9 (0.5 - 1.4) and 2.1 (1.3 - 3.4) µmol/L.

### Associations of CSF- and serum-TMAO concentrations

**Figure 1:**
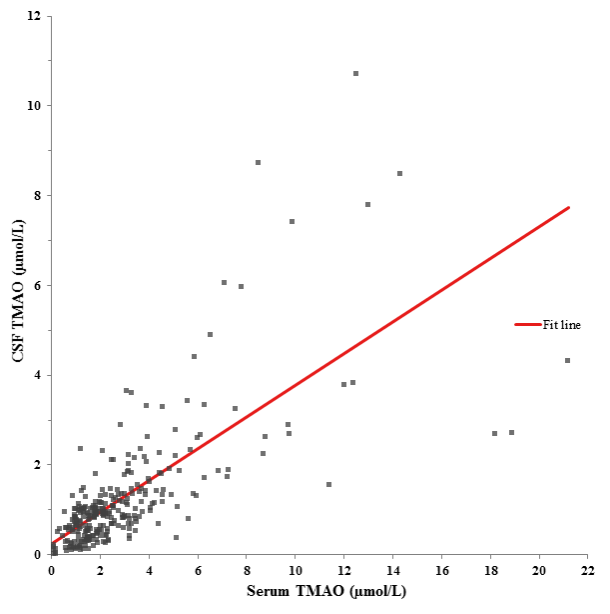
Univariate regression model between trimethylamine-N-oxide (TMAO) in human cerebrospinal fluid (CSF) and serum. ß-coefficient = 0.675, p < 0.001.

**Figure 2:**
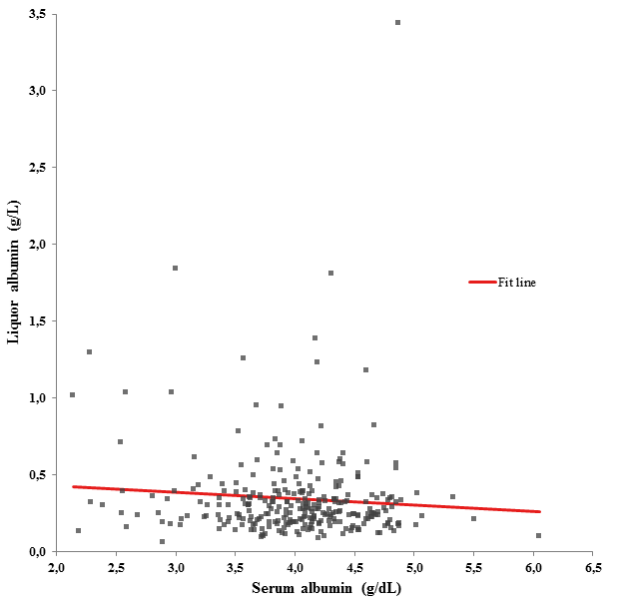
Univariate regression model between albumin in human cerebrospinal fluid (CSF) and serum. ß-coefficient = -0.081, p = 0.171.

CSF TMAO levels were positively correlated with serum TMAO levels (ρ = 0.709, p < 0.001). The univariate regression model for CSF TMAO is presented in Figure 1(Fig. 1). Serum TMAO showed a statistically relevant influence on CSF TMAO (ß-coefficient = 0.675; p < 0.001). Figure 2(Fig. 2) shows the regression line between liquor and serum albumin (ß-coefficient = -0.081, p = 0.171).

### CSF/serum TMAO and albumin quotient

**Figure 3:**
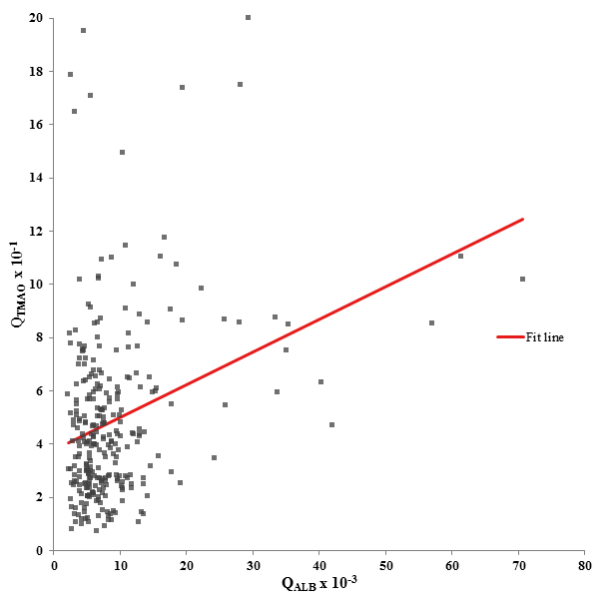
Univariate regression model between the cerebrospinal fluid (CSF)/serum trimethylamine-N-oxide (TMAO) quotient (Q_TMAO_) and the CSF/serum albumin quotient (Q_ALB_). ß-coefficient = 0.312, p < 0.001.

**Figure 4:**
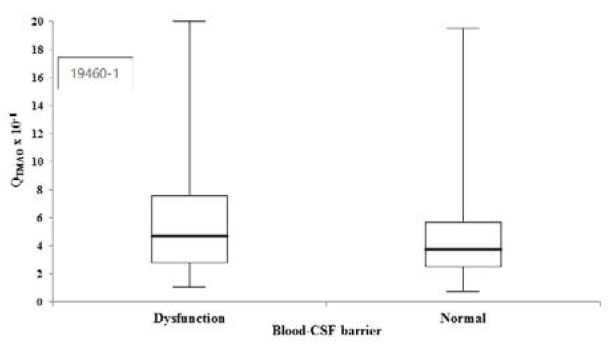
Trimethylamine-N-oxide (TMAO) and blood-cerebrospinal fluid (CSF) barrier dysfunction. Box-and-whisker plots of CSF/serum TMAO quotient (Q_TMAO_) comparisons between 117 and 173 individuals with and without blood-CSF barrier dysfunction (p = 0.002). The central boxes represent the 25^th^ to 75^th^ percentile range. The lines inside the boxes show the median value for each group. Minimum and maximum are indicated as whiskers with end caps.

The univariate regression model between the Q_TMAO _and the Q_ALB _is illustrated in Figure 3(Fig. 3). The Q_ALB _showed a statistically relevant influence on the Q_TMAO _(ß-coefficient = 0.312; p < 0.001). As shown in Figure 4(Fig. 4), 117 patients with blood-CSF barrier dysfunction had significantly higher median (Q1 - Q3) Q_TMAO _values (4.7 (2.8 - 7.5) vs. 3.8 (2.5 - 5.7) x 10^-1^, p = 0.002) compared to 173 individuals with normal blood-CSF barrier function. Raw data are provided in Supplementary Table 1.

## Discussion

In the present study, possible CSF and serum TMAO associations were assessed at the blood-CSF barrier in 290 liquor/serum pairs obtained from clinical routine. It was hypothesized that TMAO could cross the blood-CSF barrier. This aspect has never been demonstrated *in vivo.* The CSF TMAO levels were positively correlated with serum TMAO levels (ρ = 0.709, p < 0.001) and in the linear regression model serum TMAO showed a strong influence on CSF TMAO (ß-coefficient = 0.675; p < 0.001).

In comparison, Del Rio et al. measured for the first-time CSF TMAO in 58 subjects with diagnostic lumbar punctures (Del Rio et al., 2017[4]). Unfortunately, corresponding serum samples were not available, and correlation analysis between biological fluids lacked in the study design (Del Rio et al., 2017[4]). A recent work, based on different human micro-physiological systems, hypothesized that TMAO could have the potential to cross the blood-CSF barrier, but could not prove TMAO penetration (Vernetti et al., 2017[20]). 

The transport of molecules across the blood-CSF barrier is regulated by passive diffusion (e.g. albumin, immunglobulins) or facilitated by active transporters (e.g. glucose, drugs) (Tumani et al., 2017[18]). Herein, we observed a significant association between the Q_ALB _and the Q_TMAO_ (ß-coefficient = 0.312; p < 0.001). These results indicate, that the small molecule TMAO may cross the blood-CSF barrier via passive diffusion. Small molecules have been shown to penetrate the blood-CSF barrier into a much larger number compared to large molecules (Felgenhauer, 1974[6]). Since the expression of various transport proteins of the blood-CSF barrier is well known (Yasuda et al., 2013[24]), an additional active transport mechanism, which contributes to the TMAO permeation, might also be possible. Furthermore, a de novo synthesis of TMAO in the brain cannot be completely ruled out, because human FMO1 - 5 are also expressed in the adult brain (Del Rio et al., 2017[4]; Cashman and Zhang, 2006[3]). This fact might also contribute to the fraction of TMAO detected in the CSF here.

The function and dysfunction of the blood-CSF barrier are best characterized by the Q_ALB _(Brettschneider et al., 2005[2]; Tumani et al., 2017[18]). In a previous published study, Reiber demonstrated, that the Q_ALB _is the most relevant laboratory parameter for understanding the pathological dynamics of blood-derived CSF proteins (Reiber, 2001[9]). The blood-CSF barrier function was shown to comprise manifold anatomic structures at different locations (e.g. choroid plexus, ventricular surface, circumventricular organs, caudal subarachnoid space), which must be passed by blood-derived proteins, before they finally appear in the lumbar CSF (Reiber, 2003[11]).

Herein, 117 patients with blood-CSF barrier dysfunction were found with significantly higher Q_TMAO _values compared to 173 individuals with normal blood-CSF barrier function (p = 0.002). Many neurological diseases have been shown to be accompanied by an altered blood-CSF barrier permeability (Reiber, 1994[10]; Seyfert and Faulstich, 2003[15]). This circumstance may also lead to higher TMAO concentrations in this patient setting. A recent work demonstrated higher CSF TMAO concentrations in a rather small group of 18 patients with Parkinson's disease compared to 9 controls (Sankowski et al., 2020[14]).

The major advance of the present study is the high number of diagnostic lumbar punctures obtained from neurological patients. Nevertheless, the major limitation of this retrospective analysis is that it is not possible to draw conclusions about possible links between CSF TMAO concentrations and different neurological conditions. Conducting prospective studies may contribute to this gap of knowledge in the future.

## Conclusions

Significant association of CSF and serum TMAO concentrations was observed in 290 diagnostic CSF/serum pairs of clinical routine. Q_ALB _showed a relevant influence on Q_TMAO_. Patients with blood-CSF barrier dysfunction had significantly higher CSF TMAO concentrations compared to individuals with normal blood-CSF function. 

## Conflict of interest

The authors declare that there is no conflict of interest.

## Supplementary Material

Supplementary data

## Figures and Tables

**Table 1:**
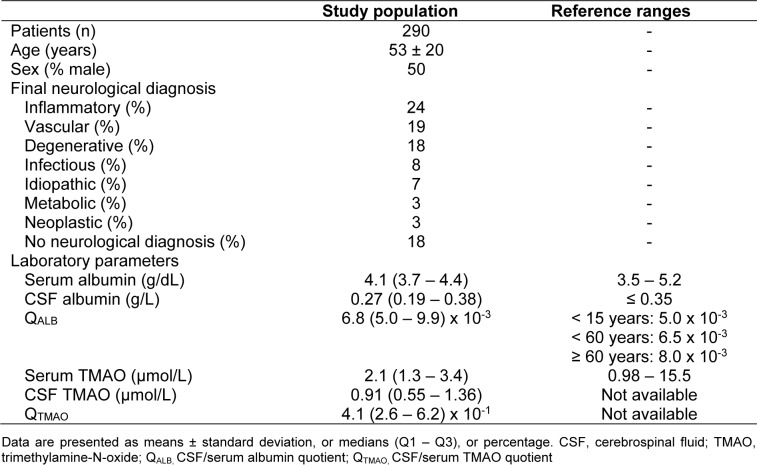
Baseline characteristics of the study population
